# Supplementary material for: Are global and specific interindividual differences in cortical thickness associated with facets of cognitive abilities, including face cognition?
Source: R Soc Open Sci. 2019 Jul 31;6(7):180857. doi: 10.1098/rsos.180857 (PMC6689650; doi:10.1098/rsos.180857)
Supplement: Behavioral performance [file rsos180857supp1.docx]

Supplement 1

Behavioral performance, means and standard deviations per task

| Task | M | SD |
| --- | --- | --- |
| WM | 103.26 | 13.26 |
| Gff1 | 16.81 | 4.85 |
| Gff2 | 14.89 | 4.48 |
| Gfv1 | 106.83 | 15.08 |
| Gfv2 | 108.99 | 15.62 |
| FWM 2b1 | 0.87 | 0.13 |
| FWM 2b2 | 0.90 | 0.13 |
| FWM 0b1 | 0.91 | 0.15 |
| FWM 0b2 | 0.96 | 0.09 |
| Frec1 | 0.65 | 0.10 |
| Frec2 | 0.66 | 0.09 |
| ER | 6.83 | 0.74 |

Note. WM – working memory, in IQ scale; Gff1 – figural task, Raven’s progressive matrices, value represents average number of correctly solved trials; Gff2 – figural task, spatial line orientation, value represents average number of correctly solved trials; Gfv1 – verbal task, oral reading recognition, in IQ scale; Gfv2 – verbal task, vocabulary comprehension, in IQ scale; FWM – working memory task with facial content in a 2-back and a 0-back condition, values represent average proportion of correctly solved trials; FRec – recognition memory of faces from the inside-scanner working memory task, values represent average proportion of correctly solved trials; ER – facial emotion recognition, value represents average number of correctly solved trials.

Supplementary material to the following article:

Meyer, K., Garzón, B., Lövdén, M., Hildebrandt, A. (2019). Are Global and Specific Interindividual Differences in Cortical Thickness Associated with Facets of Cognitive Abilities, Including Face Cognition? Royal Society Open Science.
