# Supplementary material for: Are global and specific interindividual differences in cortical thickness associated with facets of cognitive abilities, including face cognition?
Source: R Soc Open Sci. 2019 Jul 31;6(7):180857. doi: 10.1098/rsos.180857 (PMC6689650; doi:10.1098/rsos.180857)
Supplement: Table of parcel names [file rsos180857supp2.docx]

Supplement 2

Parcels from the HCP_MMP1.0 atlas, composing ROIs used in this study

| ROI | Parcel Names |
| --- | --- |
| Vis | V1, V2, V3 |
| PM | 6a, 6d, 6r, 6v |
| SPC | LIPv, VIP, AIP, MIP, 7PC, 7AL, 7Am, 7PL, 7Pm |
| dlPFC | 8C, 8Av, i6-8, s6-8, SFL, 8BL, 9p, 9a, 8Ad, p9-46v, a9-46v, 46, 9-46d |
| FFA | FFC, VVC |
| OFA | PIT, LO2, V4t |
| pSTS | TPOJ1, TPOJ2, PHT |

Note. Vis – visual cortex; PM – premotor area; SPC – superior parietal cortex; dlPFC – dorsolateral prefrontal cortex; FFA – fusiform face area; OFA – occipital face area; pSTS – posterior superior temporal sulcus.

Supplementary material to the following article:

Meyer, K., Garzón, B., Lövdén, M., Hildebrandt, A. (2019). Are Global and Specific Interindividual Differences in Cortical Thickness Associated with Facets of Cognitive Abilities, Including Face Cognition? Royal Society Open Science.
