# Supplementary material for: Are global and specific interindividual differences in cortical thickness associated with facets of cognitive abilities, including face cognition?
Source: R Soc Open Sci. 2019 Jul 31;6(7):180857. doi: 10.1098/rsos.180857 (PMC6689650; doi:10.1098/rsos.180857)
Supplement: ROI locations in MNI space [file rsos180857supp3.docx]

Supplement 3

Location of vertices with the highest probability to belong to the functionally localized core face network ROI in MNI coordinates

|  | Left | | | | Right | | | |
| --- | --- | --- | --- | --- | --- | --- | --- | --- |
|  | x | y | z | Density | x | y | z | Density |
| FFA | -43 | -58 | -25 | 213 | 44 | -67 | -23 | 277 |
| OFA | -35 | -92 | -18 | 226 | 37 | -92 | -13 | 265 |
| pSTS | -58 | -49 | 4 | 114 | 58 | -41 | 3 | 118 |
| FFA | -43 | -65 | -24 | 528 | 49 | -66 | -23 | 617 |
| OFA | -36 | -90 | -19 | 594 | 39 | -91 | -14 | 635 |
| pSTS | -58 | -50 | 5 | 317 | 57 | -43 | 3 | 302 |

*Note.* The functionally localized statistical fMRI masks for each subject were superimposed. Because the values of the statistical maps were all 1 in every vertex belonging to a mask, the density represents the total number of subjects for which the particular location in MNI space was included in their individually localized masks of FFA, OFA or pSTS.

Supplementary material to the following article:

Meyer, K., Garzón, B., Lövdén, M., Hildebrandt, A. (2019). Are Global and Specific Interindividual Differences in Cortical Thickness Associated with Facets of Cognitive Abilities, Including Face Cognition? Royal Society Open Science.
