# Supplementary material for: Are global and specific interindividual differences in cortical thickness associated with facets of cognitive abilities, including face cognition?
Source: R Soc Open Sci. 2019 Jul 31;6(7):180857. doi: 10.1098/rsos.180857 (PMC6689650; doi:10.1098/rsos.180857)
Supplement: Regression weights [file rsos180857supp4.docx]

Supplement 4

Standardized regression weights, standard errors and significance

|  | Model | Factor | Regressor | β | SE | p |
| --- | --- | --- | --- | --- | --- | --- |
| Accuracy Models as preregistered | acc1 | accG | Age | -.01 | .01 | .44 |
|  |  |  | Sex | .34 | .08 | <.001 |
|  | acc2 | accG | Age | -.01 | .01 | .65 |
|  |  |  | Sex | .38 | .08 | <.001 |
|  |  | accF | Age | -.02 | .02 | .19 |
|  |  |  | Sex | -.23 | .12 | .04 |
| CT Models left hemisphere, ROI 5 mm | CT1 L | CTG L | Age | -.05 | .01 | <.001 |
|  |  |  | Sex | .01 | .08 | .94 |
|  | CT2 L | CTG L | Age | - | - | - |
|  |  |  | Sex | - | - | - |
|  |  | CTF L | Age | - | - | - |
|  |  |  | Sex | - | - | - |
| CT Models right hemisphere, ROI 5 mm | CT1 R | CTG R | Age | -.05 | .01 | <.001 |
|  |  |  | Sex | -.17 | .08 | .05 |
|  | CT2 R | CTG R | Age | -.06 | .01 | <.001 |
|  |  |  | Sex | .02 | .08 | .82 |
|  |  | CTF R | Age | .15 | .08 | .07 |
|  |  |  | Sex | -.10 | .30 | .73 |
| Accuracy Models, modified | acc1m | accGm | Age | -.01 | .01 | .61 |
|  |  |  | Sex | .38 | .08 | <.001 |
|  | acc2m | accGm | Age | -.01 | .01 | .55 |
|  |  |  | Sex | .40 | .08 | <.001 |
|  |  | accFm | Age | .02 | .02 | .20 |
|  |  |  | Sex | -.44 | .14 | .001 |
| CT Models left hemisphere, ROI 10 mm | CT1m L | CTGm L | Age | -.04 | .01 | .003 |
|  |  |  | Sex | -.20 | .09 | .02 |
|  | CT2m L | CTGm L | Age | -.04 | .01 | .002 |
|  |  |  | Sex | -.21 | .09 | .02 |
|  |  | CTFm L | Age | .03 | .03 | .24 |
|  |  |  | Sex | .14 | .20 | .50 |
| CT Models right hemisphere, ROI 10 mm | CT1m R | CTGm R | Age | -.04 | .01 | .002 |
|  |  |  | Sex | -.21 | .09 | .017 |
|  | CT2m R | CTGm R | Age | -.04 | .01 | .001 |
|  |  |  | Sex | -.22 | .09 | .01 |
|  |  | CTFm R | Age | .11 | .07 | .13 |
|  |  |  | Sex | .28 | .32 | .38 |
| CT Models left hemisphere, ROI mask across-subject | CT1m L | CTGm L | Age | -.03 | .01 | .007 |
|  |  |  | Sex | -.22 | .09 | .01 |
|  | CT2m L | CTGm L | Age | -.04 | .01 | .001 |
|  |  |  | Sex | -.23 | .09 | .01 |
|  |  | CTFm L | Age | .03 | .02 | .09 |
|  |  |  | Sex | .04 | .15 | .80 |
| CT Models right hemisphere, ROI mask across-subject | CT1m R | CTGm R | Age | -.03 | .01 | .004 |
|  |  |  | Sex | -.19 | .09 | .03 |
|  | CT2m R | CTGm R | Age | -.04 | .01 | <.001 |
|  |  |  | Sex | -.22 | .09 | .01 |
|  |  | CTFm R | Age | .04 | .02 | .04 |
|  |  |  | Sex | .20 | .15 | .18 |
| Accuracy + CT left, ROI mask across-subject | accG + CT L | accGm | Age | -.02 | .01 | .15 |
|  |  |  | Sex | .38 | .09 | <.001 |
|  |  |  | CTGm L | -.06 | .05 | .17 |
|  |  |  | CTFm L | .26 | .09 | .003 |
|  |  | accFm | Age | -.02 | .02 | .32 |
|  |  |  | Sex | .49 | .13 | <.001 |
|  |  |  | CTGm L | .07 | .06 | .26 |
|  |  |  | CTFm L | .04 | .11 | .69 |
|  |  | CTGm L | Age | -.04 | .01 | .001 |
|  |  |  | Sex | -.23 | .09 | .01 |
|  |  | CTFm L | Age | .03 | .02 | .09 |
|  |  |  | Sex | .04 | .14 | .79 |
| Accuracy + CT right, ROI mask across-subject | accG + CT R | accGm | Age | -.02 | .01 | .16 |
|  |  |  | Sex | .35 | .09 | <.001 |
|  |  |  | CTGm R | -.05 | .05 | .31 |
|  |  |  | CTFm R | .23 | .10 | .03 |
|  |  | accFm | Age | .01 | .02 | .57 |
|  |  |  | Sex | -.50 | .13 | <.001 |
|  |  |  | CTGm R | -.05 | .07 | .45 |
|  |  |  | CTFm R | .14 | .12 | .24 |
|  |  | CTGm R | Age | -.04 | .01 | .001 |
|  |  |  | Sex | -.22 | .09 | .01 |
|  |  | CTFm R | Age | .04 | .02 | .05 |
|  |  |  | Sex | .20 | .15 | .18 |

Note. accG – General factor of performance accuracy; accF – nested factor of performance accuracy in face-related tasks. CTG – general factor of cortical thickness; CTF – nested factor of CT in face-related brain areas. L and R – refer to the left and right hemisphere. m – indicates that the model series was altered compared to the preregistered protocol and is part of the post-hoc analysis. β – standardized regression weights; SE – standard error.

Supplementary material to the following article:

Meyer, K., Garzón, B., Lövdén, M., Hildebrandt, A. (2019). Are Global and Specific Interindividual Differences in Cortical Thickness Associated with Facets of Cognitive Abilities, Including Face Cognition? Royal Society Open Science.
