# Supplementary material for: Are global and specific interindividual differences in cortical thickness associated with facets of cognitive abilities, including face cognition?
Source: R Soc Open Sci. 2019 Jul 31;6(7):180857. doi: 10.1098/rsos.180857 (PMC6689650; doi:10.1098/rsos.180857)
Supplement: Factor loadings of accuracy models [file rsos180857supp5.docx]

Supplement 5

Standardized estimates of factor loadings with standard errors for general and nested factors of accuracy – preregistered and post-hoc modified models

|  |  | Model accG | | Model accF | | | | Model accF – loadings restrained | | | |
| --- | --- | --- | --- | --- | --- | --- | --- | --- | --- | --- | --- |
|  |  | Factors | | | | | | | | | |
|  |  | accG | | accG | | accF | | accG | | accF | |
| As Preregistered | Indicator | Est | SE | Est | SE | Est | SE | Est | SE | Est | SE |
|  | WM | .487 | .036 | .479 | .036 | - | - | .479 | .036 | - | - |
|  | Gff1 | .633 | .030 | .626 | .031 | - | - | .627 | .031 | - | - |
|  | Gff2 | .528 | .033 | .533 | .033 | - | - | .533 | .033 | - | - |
|  | Gfv1 | .778 | .023 | .797 | .021 | - | - | .795 | .021 | - | - |
|  | Gfv2 | .761 | .025 | .779 | .024 | - | - | .777 | .025 | - | - |
|  | FWM2b1 | .468 | .032 | .444 | .033 | .165 | .077 | .448 | .032 | .121 | .067 |
|  | FWM2b2 | .493 | .044 | .459 | .046 | .281 | .083 | .462 | .046 | .254 | .081 |
|  | FWM0b1 | .355 | .041 | .308 | .041 | .519 | .062 | .315 | .040 | .452 | .036 |
|  | FWM0b2 | .365 | .043 | .311 | .042 | .653 | .090 | .313 | .042 | .748 | .059 |
|  | Frec1 | .216 | .035 | .211 | .036 | .033 | .060 | .211 | .036 | .048 | .043 |
|  | Frec2 | .202 | .040 | .188 | .039 | .089 | .061 | .191 | .039 | .054 | .049 |
|  | ER | .270 | .037 | .271 | .037 | .004 | .054 | .270 | .037 | .015 | .045 |

|  |  | Model accG | | Model accF | | | | Model accF – loadings restrained | | | |
| --- | --- | --- | --- | --- | --- | --- | --- | --- | --- | --- | --- |
|  |  | Factors | | | | | | | | | |
|  |  | accG | | accG | | accF | | accG | | accF | |
| Modified Accuracy Models | Indicator | Est | SE | Est | SE | Est | SE | Est | SE | Est | SE |
|  | WM | .473 | .036 | .473 | .036 | - | - | .473 | .036 | - | - |
|  | Gff1 | .621 | .031 | .622 | .031 | - | - | .622 | .031 | - | - |
|  | Gff2 | .531 | .034 | .533 | .034 | - | - | .533 | .034 | - | - |
|  | Gfv1 | .800 | .020 | .799 | .020 | - | - | .799 | .020 | - | - |
|  | Gfv2 | .783 | .024 | .785 | .024 | - | - | .785 | .024 | - | - |
|  | FWM2b1 | .427 | .032 | .428 | .032 | .045 | .053 | .429 | .032 | .095 | .044 |
|  | FWM2b2 | .442 | .045 | .445 | .045 | .156 | .052 | .444 | .045 | .097 | .045 |
|  | Frec1 | .212 | .035 | .219 | .036 | .380 | .077 | .224 | .036 | .441 | .038 |
|  | Frec2 | .189 | .039 | .205 | .039 | .576 | .108 | .204 | .039 | .500 | .041 |

Note. accG and accF – latent variables of performance accuracy in general and face-specific behavioral tasks. Indicated by the following psychometric tasks: WM – working memory; Gff1 – figural task, Raven’s progressive matrices; Gff2 – figural task, spatial line orientation; Gfv1 – verbal task, oral reading recognition; Gfv2 – verbal task, vocabulary comprehension; FWM – working memory task with facial content in a 2-back and a 0-back condition; FRec – recognition memory of faces from the inside-scanner working memory task; ER – facial emotion recognition.

Supplementary material to the following article:

Meyer, K., Garzón, B., Lövdén, M., Hildebrandt, A. (2019). Are Global and Specific Interindividual Differences in Cortical Thickness Associated with Facets of Cognitive Abilities, Including Face Cognition? Royal Society Open Science.
