# Supplementary material for: Are global and specific interindividual differences in cortical thickness associated with facets of cognitive abilities, including face cognition?
Source: R Soc Open Sci. 2019 Jul 31;6(7):180857. doi: 10.1098/rsos.180857 (PMC6689650; doi:10.1098/rsos.180857)
Supplement: Factor loadings of CT models [file rsos180857supp6.docx]

Supplement 6

Standardized estimates of factor loadings with standard errors for general and nested factors of CT – preregistered and post-hoc modified models

|  |  | |  | | General ROIs | | | | | | | | | | | | | | | | Face-Related ROIs | | | | | | | | | | |  |
| --- | --- | --- | --- | --- | --- | --- | --- | --- | --- | --- | --- | --- | --- | --- | --- | --- | --- | --- | --- | --- | --- | --- | --- | --- | --- | --- | --- | --- | --- | --- | --- | --- |
|  |  | |  | | Vis | | | | PM | | | | SPC | | | | dlPFC | | | | FFA | | | | OFA | | | | pSTS | | |  |
|  | Model | | Factor | | Est | | SE | | Est | | SE | | Est | | SE | | Est | | SE | | Est | | SE | | Est | | SE | | Est | | SE |  |
| ROI 5 mm as preregistered | CTG L | | CTG | | .515 | | .053 | | .906 | | .014 | | .755 | | .022 | | .865 | | .014 | | .274 | | .043 | | .245 | | .032 | | .327 | | .045 |  |
|  | CTG + CTF L | | CTG | | - | | - | | - | | - | | - | | - | | - | | - | | - | | - | | - | | - | | - | | - |  |
|  |  |  | CTF | | - | | - | | - | | - | | - | | - | | - | | - | | - | | - | | - | | - | | - | | - |  |
|  | CTG R | | CTG | | .544 | | .033 | | .838 | | .018 | | .785 | | .017 | | .791 | | .021 | | .514 | | .031 | | .523 | | .031 | | .651 | | .026 |  |
|  | CTG + GTF R | | CTG | | .475 | | .033 | | .887 | | .014 | | .752 | | .019 | | .829 | | .017 | | .247 | | .039 | | .256 | | .037 | | .267 | | .037 |  |
|  |  |  | CTF | | - | | - | | - | | - | | - | | - | | - | | - | | .125 | | .085 | | .224 | | .121 | | .069 | | .073 |  |
|  |  |  | | General ROIs | | | | | | | | | | | | | | | | Face-Related ROIs | | | | | | | | | | | | |
|  |  |  | | Vis | | | | PM | | | | SPC | | | | dlPFC | | | | FFA | | | | OFA | | | | pSTS | | | | |
|  | Model | Factor | | Est | | SE | | Est | | SE | | Est | | SE | | Est | | SE | | Est | | SE | | Est | | SE | | Est | | SE | | |
| ROI 10 mm | CTG L | CTG | | .598 | | .047 | | .782 | | .030 | | .845 | | .017 | | .739 | | .030 | | .379 | | .042 | | .359 | | .037 | | .482 | | .046 | | |
|  | CTG + CTF L | CTG | | .594 | | .048 | | .784 | | .031 | | .848 | | .018 | | .741 | | .031 | | .377 | | .043 | | .352 | | .038 | | .480 | | .046 | | |
|  |  | CTF | | - | | - | | - | | - | | - | | - | | - | | - | | .496 | | .407 | | .254 | | .203 | | .024 | | .073 | | |
|  | CTG R | CTG | | .543 | | .031 | | .769 | | .023 | | .848 | | .020 | | .715 | | .025 | | .310 | | .036 | | .374 | | .035 | | .401 | | .035 | | |
|  | CTG + GTF R | CTG | | .540 | | .032 | | .773 | | .023 | | .844 | | .021 | | .720 | | .025 | | .314 | | .037 | | .390 | | .036 | | .406 | | .036 | | |
|  |  | CTF | | - | | - | | - | | - | | - | | - | | - | | - | | .086 | | .095 | | .266 | | .175 | | .095 | | .069 | | |
|  |  | |  | | General ROIs | | | | | | | | | | | | | | | | Face-Related ROIs | | | | | | | | | | |  |
|  |  | |  | | Vis | | | | PM | | | | SPC | | | | dlPFC | | | | FFA | | | | OFA | | | | pSTS | | |  |
|  | Model | | Factor | | Est | | SE | | Est | | SE | | Est | | SE | | Est | | SE | | Est | | SE | | Est | | SE | | Est | | SE |  |
| ROI mask across-subject | CTG L | | CTG | | .671 | | .042 | | .735 | | .033 | | .790 | | .019 | | .685 | | .033 | | .628 | | .045 | | .661 | | .032 | | .767 | | .029 |  |
|  | CTG + CTF L | | CTG | | .634 | | .076 | | .758 | | .063 | | .815 | | .037 | | .709 | | .051 | | .571 | | .075 | | .604 | | .055 | | .725 | | .066 |  |
|  |  |  | CTF | | - | | - | | - | | - | | - | | - | | - | | - | | .395 | | .074 | | .348 | | .079 | | .213 | | .055 |  |
|  | CTG R | | CTG | | .618 | | .029 | | .743 | | .023 | | .783 | | .022 | | .679 | | .028 | | .556 | | .031 | | .580 | | .031 | | .701 | | .024 |  |
|  | CTG + GTF R | | CTG | | .579 | | .041 | | .758 | | .035 | | .805 | | .036 | | .699 | | .038 | | .506 | | .041 | | .527 | | .039 | | .660 | | .037 |  |
|  |  |  | CTF | | - | | - | | - | | - | | - | | - | | - | | - | | .384 | | .074 | | .338 | | .070 | | .261 | | .058 |  |

*Note.* CTG – general factor of cortical thickness; CTF – nested factor of CT in face-related brain areas. CTG – general factor of CT; CTF – nested factor of CT in face-related brain areas. Indicated by local CT in brain regions: Vis – primary and secondary visual cortices; PM – premotor cortex; SPC – superior parietal cortex; DLPFC – dorsolateral prefrontal cortex; FFA – fusiform face area; OFA – occipital face area; pSTS – posterior superior temporal sulcus.

Supplementary material to the following article:

Meyer, K., Garzón, B., Lövdén, M., Hildebrandt, A. (2019). Are Global and Specific Interindividual Differences in Cortical Thickness Associated with Facets of Cognitive Abilities, Including Face Cognition? Royal Society Open Science.
