# Supplementary material for: Are global and specific interindividual differences in cortical thickness associated with facets of cognitive abilities, including face cognition?
Source: R Soc Open Sci. 2019 Jul 31;6(7):180857. doi: 10.1098/rsos.180857 (PMC6689650; doi:10.1098/rsos.180857)
Supplement: Factor loadings of brain and behavior models [file rsos180857supp7.docx]

Supplement 7

Standardized estimates of factor loadings with standard errors for general and nested factors of accuracy and CT – structural model of brain-behavior relationship, modified post-hoc, across-subject ROI

|  | Model accCT left-hemisphere | | | | | | | | Model accCT right-hemisphere | | | | | | | |
| --- | --- | --- | --- | --- | --- | --- | --- | --- | --- | --- | --- | --- | --- | --- | --- | --- |
|  | accG | | accF | | CTG L | | CTF L | | accG | | accF | | CTG R | | CTF R | |
| Indicator | Est | SE | Est | SE | Est | SE | Est | SE | Est | SE | Est | SE | Est | SE | Est | SE |
| WM | 0.47 | 0.04 | - | - | - | - | - | - | 0.47 | 0.04 | - | - | - | - | - | - |
| Gff1 | 0.62 | 0.03 | - | - | - | - | - | - | 0.62 | 0.03 | - | - | - | - | - | - |
| Gff2 | 0.53 | 0.03 | - | - | - | - | - | - | 0.53 | 0.03 | - | - | - | - | - | - |
| Gfv1 | 0.80 | 0.02 | - | - | - | - | - | - | 0.80 | 0.02 | - | - | - | - | - | - |
| Gfv2 | 0.79 | 0.02 | - | - | - | - | - | - | 0.79 | 0.02 | - | - | - | - | - | - |
| FWM2b1 | 0.43 | 0.03 | -0.10 | 0.04 | - | - | - | - | 0.43 | 0.03 | 0.099 | 0.05 | - | - | - | - |
| FWM2b2 | 0.44 | 0.05 | -0.10 | 0.04 | - | - | - | - | 0.44 | 0.05 | 0.10 | 0.05 | - | - | - | - |
| Frec1 | 0.23 | 0.04 | -0.44 | 0.04 | - | - | - | - | 0.21 | 0.04 | 0.44 | 0.04 | - | - | - | - |
| Frec2 | 0.21 | 0.04 | -0.50 | 0.04 | - | - | - | - | 0.19 | 0.04 | 0.50 | 0.04 | - | - | - | - |
| Vis | - | - | - | - | 0.64 | 0.05 | - | - | - | - | - | - | 0.59 | 0.03 | - | - |
| PM | - | - | - | - | 0.77 | 0.03 | - | - | - | - | - | - | 0.77 | 0.02 | - | - |
| SPC | - | - | - | - | 0.83 | 0.02 | - | - | - | - | - | - | 0.82 | 0.02 | - | - |
| dlPFC | - | - | - | - | 0.72 | 0.03 | - | - | - | - | - | - | 0.71 | 0.02 | - | - |
| FFA | - | - | - | - | 0.58 | 0.05 | 0.41 | 0.06 | - | - | - | - | 0.51 | 0.03 | 0.40 | 0.06 |
| OFA | - | - | - | - | 0.61 | 0.04 | 0.32 | 0.07 | - | - | - | - | 0.54 | 0.03 | 0.32 | 0.06 |
| pSTS | - | - | - | - | 0.73 | 0.04 | 0.25 | 0.06 | - | - | - | - | 0.67 | 0.03 | 0.29 | 0.06 |

*Note.* accG and accF – latent variables accounting for performance accuracy in general and face-specific behavioral tasks. Indicators are: WM – working memory; Gff1 – figural task, progressive matrices; Gff2 – figural task, spatial line orientation; Gfv1 – verbal task, oral reading recognition; Gfv2 – verbal task, vocabulary comprehension; FWM – working memory task with facial content in a 0-back and a 2-back condition; FRec – recognition memory of faces from the inside-scanner working memory task; ER – facial emotion recognition. CTG and CTF – global and face-specific CT. CT indicators: Vis – primary and secondary visual cortices; PM – premotor cortex; SPC – superior parietal cortex; DLPFC – dorsolateral prefrontal cortex; FFA – fusiform face area; OFA – occipital face area; pSTS – posterior superior temporal sulcus.

Supplementary material to the following article:

Meyer, K., Garzón, B., Lövdén, M., Hildebrandt, A. (2019). Are Global and Specific Interindividual Differences in Cortical Thickness Associated with Facets of Cognitive Abilities, Including Face Cognition? Royal Society Open Science.
