# Supplementary material for: Are global and specific interindividual differences in cortical thickness associated with facets of cognitive abilities, including face cognition?
Source: R Soc Open Sci. 2019 Jul 31;6(7):180857. doi: 10.1098/rsos.180857 (PMC6689650; doi:10.1098/rsos.180857)
Supplement: Correlations between CT measures acquired using different ROIs [file rsos180857supp8.docx]

Supplement 8


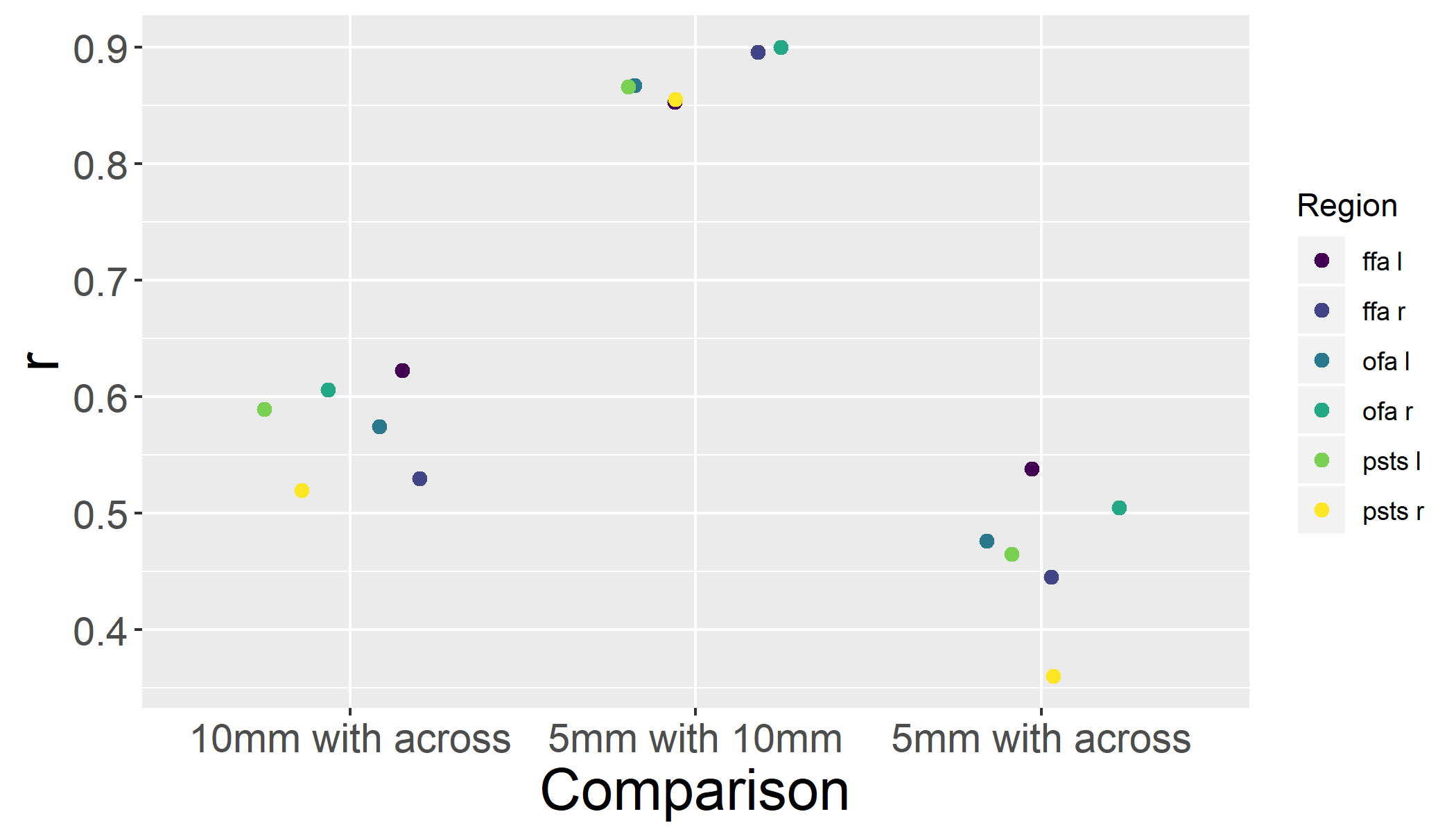
Correlations between CT measures acquired using different ROI sizes

Note. Colors denote different brain regions. ffa – fusiform face area; ofa – occipital face area; psts – posterior superior temporal sulcus; L/r – areas belonging to the left or right hemisphere, respectively.

Supplementary material to the following article:

Meyer, K., Garzón, B., Lövdén, M., Hildebrandt, A. (2019). Are Global and Specific Interindividual Differences in Cortical Thickness Associated with Facets of Cognitive Abilities, Including Face Cognition? Royal Society Open Science.
