# Supplementary material for: Are global and specific interindividual differences in cortical thickness associated with facets of cognitive abilities, including face cognition?
Source: R Soc Open Sci. 2019 Jul 31;6(7):180857. doi: 10.1098/rsos.180857 (PMC6689650; doi:10.1098/rsos.180857)
Supplement: Results of multiple group analysis [file rsos180857supp9.docx]

Supplement 8

Fit statistics of multiple group models testing for invariance across gender groups

| Models | | Wald-Test | Δdf | *p* | CFI | RMSEA | SRMR |
| --- | --- | --- | --- | --- | --- | --- | --- |
| accG + accF | Configural |  |  |  | .93 | .06 | .05 |
|  | Metric | 6.67 | 11 | .82 | .94 | .05 | .04 |
|  | Strict | 23.17 | 9 | <.01 | .93 | .06 | .05 |
| CTG + CTF L | Configural |  |  |  | .96 | .09 | .05 |
|  | Metric | 6.50 | 10 | .77 | .97 | .08 | .06 |
|  | Strict | 8.91 | 7 | .26 | .97 | .08 | .06 |
| CTG + CTF R | Configural |  |  |  | .93 | .11 | .05 |
|  | Metric | 15.87 | 10 | .10 | .93 | .10 | .07 |
|  | Strict | 10.56 | 7 | .16 | .93 | .09 | .07 |
| Acc + CT L | Configural |  |  |  | .94 | .05 | .05 |
|  | Metric | 14.32 | 21 | .86 | .95 | .05 | .05 |
|  | Strict | 38.11 | 16 | <.01 | .95 | .05 | .06 |
| Acc + CT R | Configural |  |  |  | .92 | .06 | .06 |
|  | Metric | 23.31 | 21 | .33 | .93 | .05 | .06 |
|  | Strict | 38.27 | 16 | <.01 | .92 | .05 | .06 |

*Note.* Δχ2 (Δ*df*): Nested models were compared using the Wald-test. accG – General factor of performance accuracy; accF – nested factor of face-related performance; CTG – general factor of CT (CT); CTF – nested factor of CT in face-related brain areas; acc + CT – these models represent a combination of the respective accuracy and CT models (figure 3); R – indicates that right-hemispheric ROIs were modeled; L – indicates the use of left-hemispheric ROIs.

Supplementary material to the following article:

Meyer, K., Garzón, B., Lövdén, M., Hildebrandt, A. (2019). Are Global and Specific Interindividual Differences in Cortical Thickness Associated with Facets of Cognitive Abilities, Including Face Cognition? Royal Society Open Science.
