# Supplementary material for: Are global and specific interindividual differences in cortical thickness associated with facets of cognitive abilities, including face cognition?
Source: R Soc Open Sci. 2019 Jul 31;6(7):180857. doi: 10.1098/rsos.180857 (PMC6689650; doi:10.1098/rsos.180857)
Supplement: Follow-up models separating performance and CT indicators [file rsos180857supp10.docx]

Supplement 9

Fit statistics and regression weights for follow-up model series separating general and face-related indicators

| Latent DV | Regressor | *χ2* (df) | CFI | RMSEA | SRMR | *β* | SE | *p* |
| --- | --- | --- | --- | --- | --- | --- | --- | --- |
| AccG | CTG left | 175.03 (39) | .95 | .06 | .04 | -.08 | .05 | .09 |
|  | CTG right | 179.18 (39) | .94 | .07 | .04 | -.06 | .05 | .22 |
|  | CTF left | 118.48 (31) | .95 | .06 | .04 | .06 | .05 | .20 |
|  | CTF right | 130.67 (31) | .93 | .06 | .04 | .05 | .06 | 34 |
| AccF | CTG left | 103.5 (31) | .96 | .05 | .04 | .11 | .06 | .06 |
|  | CTG right | 102.30 (31) | .96 | .05 | .04 | -.08 | .06 | .16 |
|  | CTF left | 48.6 (24) | .97 | .04 | .04 | -.04 | .06 | .49 |
|  | CTF right | 48.5 (24) | .96 | .04 | .04 | .06 | .07 | .40 |

*Note.* accG – General factor of performance accuracy; accF – nested factor of face-related performance; CTG – general factor of CT; CTF – nested factor of CT in face-related brain areas.

Supplementary material to the following article:

Meyer, K., Garzón, B., Lövdén, M., Hildebrandt, A. (2019). Are Global and Specific Interindividual Differences in Cortical Thickness Associated with Facets of Cognitive Abilities, Including Face Cognition? Royal Society Open Science.
